# Supplementary material for: Obesity Affects the Proliferative Potential of Equine Endometrial Progenitor Cells and Modulates Their Molecular Phenotype Associated with Mitochondrial Metabolism
Source: Cells. 2022 Apr 24;11(9):1437. doi: 10.3390/cells11091437 (PMC9100746; doi:10.3390/cells11091437)
Supplement: Supplementary file 1 [file cells-11-01437-s001.zip › Figure S1.pdf]

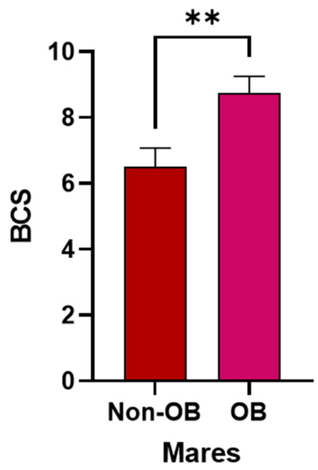

Figure S1. The results of body condition score (BCS) examination performed on live mares for proper classification of animals. Columns with bars represent mean ± SD. * p-value < 0.05, ** p-value < 0.01.
